# Resveratrol Modulates Bone Metabolism Through SIRT1‐Mediated NF‐κB Inhibition: Molecular Mechanisms and Therapeutic Implications

**DOI:** 10.1002/cbf.70334

**Published:** 2026-11-01

**Authors:** Seyyed Hossein Khatami, Faezeh Gouhari, Alireza Manafi‐Rasi, Maryam Fallah, Sajad Ehtiati, Ahmad Reza Ahmadi Abdashti, Mohsen Ariannik, Shahin Talebi, Diba Rafienejad, Mina Fakhrzadegan, Parasta Heidari, Reza Zandi

**Affiliations:** ^1^ Musculoskeletal Injuries Research Center Shahid Beheshti University of Medical Sciences Tehran Iran; ^2^ Department of Orthopedics, Imam Hossein Educational Hospital Shahid Beheshti University of Medical Sciences Tehran Iran; ^3^ Department of Clinical Biochemistry, School of Medicine Shahid Beheshti University of Medical Sciences Tehran Iran; ^4^ Center for Genomics and Personalized Health Queensland University Brisbane Australia; ^5^ School of Biomedical Sciences, Faculty of Health Queensland University of Technology Brisbane Australia

**Keywords:** bone metabolism, inflammation, NF‐κB, osteoblast, osteoclast, osteoporosis, oxidative stress, RANKL, resveratrol, SIRT1

## Abstract

Osteoporosis and associated skeletal pathologies impose a staggering global health burden, disproportionately impacting elderly populations and postmenopausal women. At the root of excessive bone resorption and stalled osteogenesis lie persistent oxidative stress and chronic inflammation. Enter resveratrol, a naturally occurring polyphenol derived from red wine and grapes, which has surfaced as a compelling therapeutic candidate for skeletal preservation owing to its robust antioxidant and anti‐inflammatory capacities. Herein, we evaluate the molecular pathways through which resveratrol governs bone metabolism, focusing sharply on SIRT1‐driven suppression of NF‐κB signaling. Integrating findings across cell cultures, animal models, and clinical trials yields a unified perspective on these osteoprotective mechanisms. Dual benefits define resveratrol's impact on skeletal architecture: it stimulates osteoblast differentiation and mineralization while suppressing osteoclastogenesis and bone resorption. Primarily, these outcomes stem from the activation of SIRT1, a NAD + ‐dependent deacetylase acting as a negative regulator of NF‐κB transcription. Through SIRT1‐mediated deacetylation of the NF‐κB p65 subunit, the transcription of pro‐inflammatory cytokines including IL‐1β, TNF‐α, and IL‐6 diminishes, subsequently dampening RANKL‐driven osteoclastogenesis. Simultaneously, through the SIRT1‐dependent engagement of Nrf2/ARE pathways, antioxidant defenses are bolstered, sparing bone cells from oxidative stress‐induced apoptosis. Substantial preclinical promise notwithstanding, hurdles persist regarding both systemic bioavailability and the standardization of ideal dosing protocols for bone preservation. This review provides a mechanistic framework to evaluate resveratrol as a viable nutraceutical modality for the prevention and management of osteoporosis.

## Key Points

- Resveratrol may support bone formation and limit bone resorption through complementary molecular mechanisms.
- SIRT1‐mediated inhibition of NF‐κB signaling may attenuate inflammation‐associated osteoclastogenesis.
- Nrf2/ARE pathway activation may strengthen antioxidant defenses in bone cells.
- Clinical translation remains limited by bioavailability, dose variability, and insufficient long‐term evidence.
- Further clinical studies are needed to establish the skeletal benefits and therapeutic applicability of resveratrol.

## 1 Introduction

Continuous remodeling characterizes bone as a dynamic tissue, relying on the synchronized actions of matrix‐synthesizing osteoblasts and bone‐resorbing osteoclasts [1, 2]. Skeletal integrity, calcium homeostasis, and structural adaptation to mechanical loads all hinge on this delicate cellular equilibrium. Whenever this balance falters, pathological skeletal conditions emerge, most prominently osteoporosis a disorder defined by declining bone mineral density (BMD) and heightened fracture vulnerability [3].

Roughly 200 million individuals globally suffer from osteoporosis, making it a formidable international health crisis where postmenopausal women face extreme vulnerability driven by estrogen deficiency [4]. Economic tolls remain severe, with annual expenditures surpassing $19 billion within the United States alone [5]. Standard clinical interventions generally rely on anabolic treatments like teriparatide alongside antiresorptive drugs such as denosumab and bisphosphonates. Yet, these pharmaceutical options often carry troubling complications, including cardiovascular risks, atypical femoral fractures, and osteonecrosis of the jaw, which collectively underscore an urgent demand for safer alternative strategies [6].

Dietary‐derived natural compounds have increasingly attracted scientific focus as viable bone‐protective alternatives in recent years. Prominent among them is resveratrol (trans‐3,5,4′‐trihydroxystilbene), a rich polyphenolic constituent found within peanuts, berries, grapes, and red wine [7]. Initially celebrated for explaining the cardioprotective nuances of the “French Paradox,” this molecule has since displayed diverse physiological benefits spanning anti‐aging, antioxidant, and anti‐inflammatory properties [8].

Numerous molecular targets govern the biological activity of resveratrol, with silent mating type information regulation 2 homolog 1 (SIRT1) serving as a central mediator [9]. As a NAD + ‐dependent protein deacetylase within the sirtuin family, SIRT1 oversees an array of cellular functions, encompassing metabolism, inflammation, stress adaptation, and cellular senescence [10]. Particularly critical to skeletal biology, SIRT1 exerts a negative regulatory effect on nuclear factor‐kappa B (NF‐κB), a master transcriptional regulator fueling inflammation and osteoclastogenesis [11].

Herein, we deliver an in‐depth exploration of the molecular pathways driving resveratrol's influence on bone metabolism, concentrating heavily on the SIRT1‐NF‐κB signaling axis. Evidence compiled from human clinical investigations, animal models, and cellular assays is evaluated to appraise the therapeutic capacity of resveratrol against osteoporosis. Finally, we address persistent hurdles tied to its systemic bioavailability alongside innovative approaches designed to optimize its clinical translation for skeletal disorders.

## 2 Resveratrol Chemistry Sources and Bioavailability

Constituting a key member of the stilbene family of polyphenols, resveratrol (3,5,4′‐trihydroxy‐trans‐stilbene) features a distinctive chemical framework built upon two aromatic rings linked through a central ethylene bridge [12, 13]. Geometric isomerism yields two distinct configurations: cis‐resveratrol and trans‐resveratrol. While biological potency and chemical stability favor the trans‐isomer, photosensitivity to ultraviolet light or thermal exposure triggers reversible conversion into the less active cis‐counterpart a crucial consideration for handling and storage protocols.

Directly driving its robust antioxidant capacity, the polyphenol core structure enables efficient free radical scavenging and transition metal ion chelation to counteract oxidative stress [14, 15]. Specific positioning of its hydroxyl groups also promotes critical hydrogen bonding with diverse enzymatic targets and proteins, underpinning multifaceted pharmacological actions like cardioprotection and anti‐inflammatory responses. Glycosylated variants like piceid also occur naturally, altering solubility and bioavailability profiles while potentially acting as metabolic reservoirs that liberate active resveratrol via gut microbiota hydrolysis.

Plants synthesize resveratrol as a phytoalexin, deploying it defensively against environmental insults such as ultraviolet irradiation, mechanical injury, and fungal attack [16, 17]. Dietary exposure remains correspondingly localized. Grapes (Vitis vinifera) and derived wines serve as primary sources, accumulating the compound predominantly within the fruit skin as part of this natural defense mechanism. Rapid, extensive metabolic clearance severely restricts oral bioavailability in human subjects despite immense therapeutic promise [18, 19]. Extensive first‐pass metabolism occurs within the intestinal mucosa and liver immediately after oral ingestion, driven largely by sulfation and glucuronidation pathways [20, 21]. Resulting sulfate and glucuronide conjugates consequently circulate at plasma levels vastly exceeding the parent drug. Although peak plasma concentrations typically manifest between 0.5 and 2 h post‐administration [22, 23], a brief 1 to 3‐h elimination half‐life demands either sustained‐release delivery systems or frequent dosing regimens to sustain effective therapeutic thresholds.

Preclinical data nevertheless indicate that biological activity may persist within these metabolites, allowing chronic dosing schedules to achieve sufficient systemic tissue accumulation for therapeutic outcomes. Advanced drug delivery approaches currently combat these pharmacokinetic hurdles through liposomal encapsulation, nanoparticle formulations, piperine co‐administration as a bioavailability enhancer, and the design of structural analogs possessing enhanced metabolic stability [24, 25].

Exhibiting a harmonious dual role in skeletal dynamics, resveratrol simultaneously stimulates matrix deposition and osteoblast activity while checking osteoclast differentiation and bone resorption. These synchronized actions ultimately elevate BMD, cementing its candidacy as a valuable therapeutic asset for skeletal preservation [26, 27] (Figure 1).

**Figure 1:**
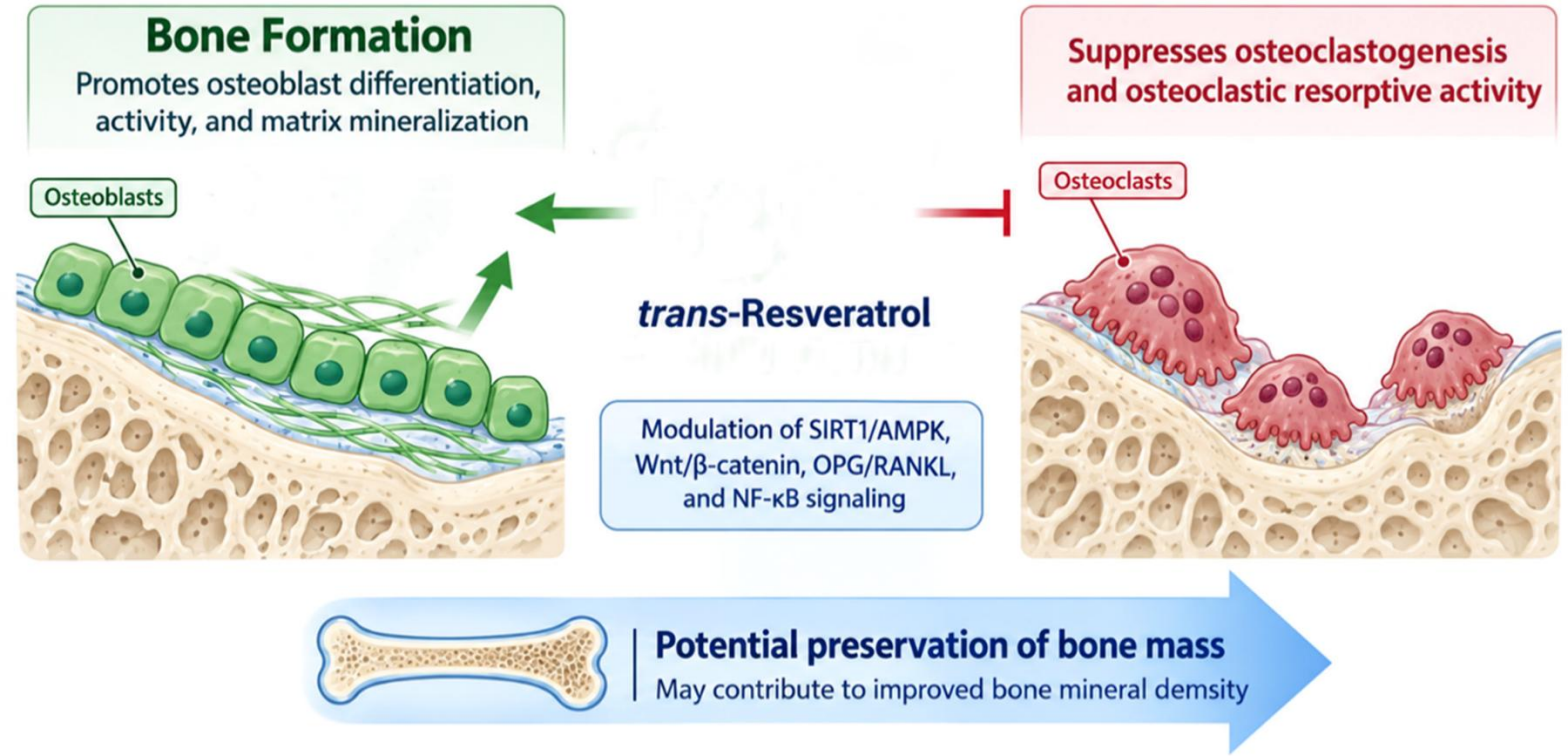
Effects of resveratrol on bone remodeling. The chemical structure of resveratrol (trans‑3,5,4′‑trihydroxystilbene) is shown at the center. Resveratrol enhances osteoblast activity and promotes bone matrix deposition, thereby supporting bone formation (left, green arrows). Concurrently, it suppresses osteoclast differentiation and bone resorption, reducing bone breakdown (right, red arrows). Through these coordinated actions, resveratrol contributes to improved bone mineral density (BMD) and a favorable balance between bone formation and resorption (Schematic created by the authors using vector graphic software).

## 3 Resveratrol's Activation of SIRT1 and Subsequent Inhibition of NF‐κB Signaling

Anchored in a precise molecular cascade, the osteoprotective properties of resveratrol initiate with the engagement of the NAD + ‐dependent protein deacetylase SIRT1, which in turn directs the inhibition of the NF‐κB signaling pathway. Acting as a master coordinator, this biochemical axis successfully reestablishes the critical equilibrium between osteoblast‐mediated bone formation and osteoclast‐driven resorption (Figure 2).

**Figure 2:**
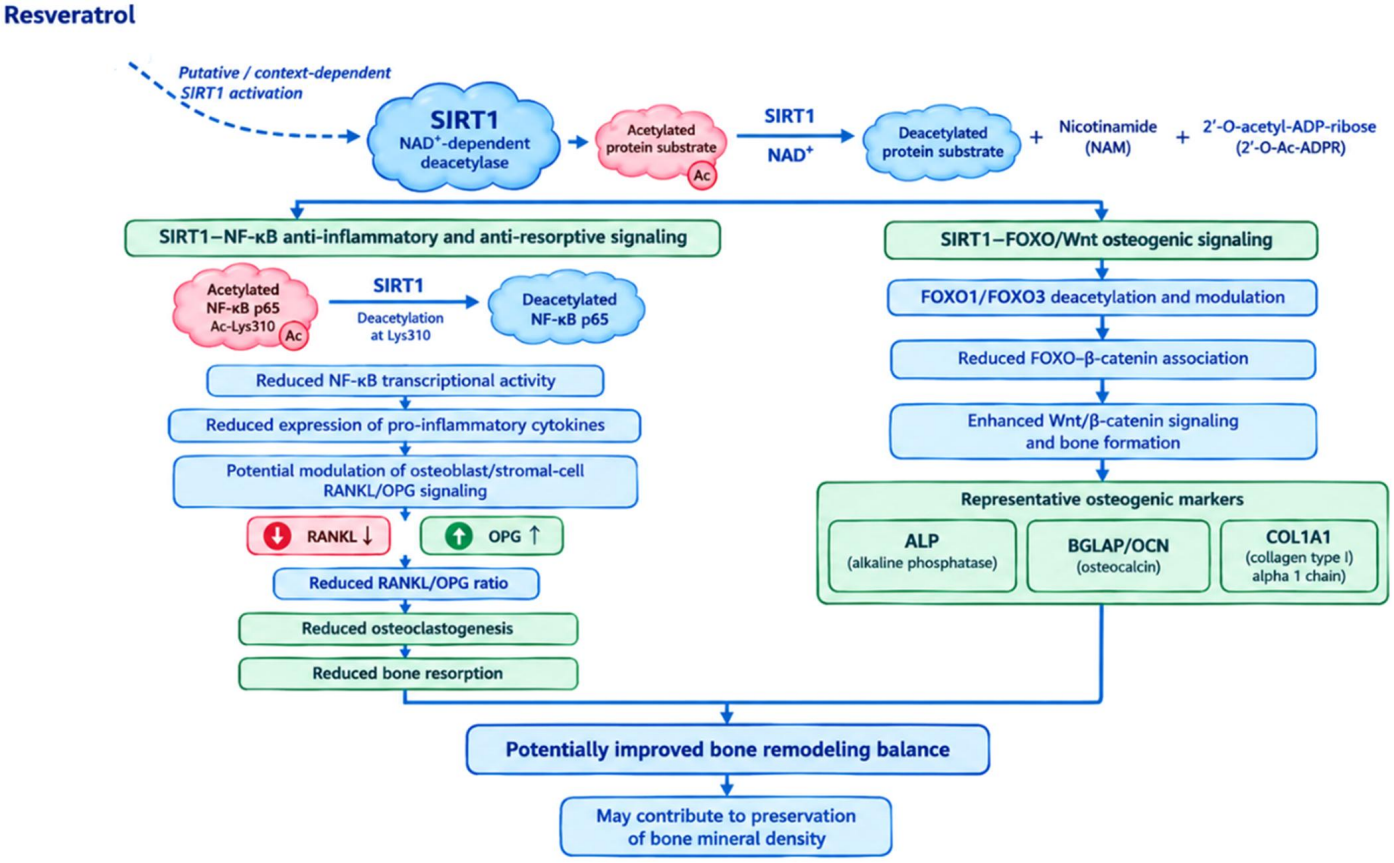
Proposed mechanisms by which resveratrol‐mediated SIRT1 activation may influence bone remodeling. Resveratrol is proposed to enhance the activity of the NAD^+^‐dependent deacetylase SIRT1 in a context‐dependent manner. Activated SIRT1 deacetylates multiple protein substrates, generating nicotinamide (NAM) and 2′‐O‐acetyl‐ADP‐ribose as reaction products. Two major downstream pathways are illustrated. On the anti‐resorptive side, SIRT1 deacetylates NF‐κB p65 (Lys310), reducing NF‐κB transcriptional activity and the expression of pro‐inflammatory cytokines. This may decrease the RANKL/OPG ratio through reduced RANKL and increased OPG expression, thereby suppressing osteoclastogenesis and bone resorption. On the osteogenic side, SIRT1‐mediated deacetylation of FOXO1/FOXO3 is proposed to reduce FOXO‐β‐catenin association and enhance Wnt/β‐catenin signaling, promoting osteoblast differentiation and the expression of osteogenic markers, including alkaline phosphatase (ALP), osteocalcin (BGLAP/OCN), and collagen type I alpha 1 (COL1A1). These mechanisms may improve bone remodeling balance and contribute to the preservation of bone mineral density. However, the proposed pathways and downstream skeletal effects are context‐dependent, and their clinical relevance in humans remains to be established (Schematic created by the authors using vector graphic software).

Understanding this mechanistic framework demands close scrutiny of resveratrol‐induced conformational shifts, the precise biochemistry governing NF‐κB suppression, and the expansive regulatory web shaped by this molecular dialog. Within skeletal pathology particularly age‐related disorders like osteoporosis physiological bone homeostasis relies heavily on SIRT1, casting its activation by resveratrol as a central motif across numerous investigations [28, 29].

Allosteric alterations follow resveratrol binding to SIRT1, driving tighter association with peptide substrates and robustly enhancing deacetylase efficiency. X‐ray crystallography reveals a ternary complex housing three resveratrol molecules, where a pair mediates critical contacts between the enzyme and its peptide substrate [28]. Crucially, this activation operates selectively rather than universally; resveratrol governs deacetylation in a substrate‐dependent manner, boosting certain proteins while inhibiting others to establish a nuanced, targeted regulatory profile rather than a blunt on‐off switch [30].

Within skeletal metabolism, the premier downstream consequence of SIRT1 activation is the potent suppression of NF‐κB a master transcriptional hub driving inflammatory responses and exerting a doubly detrimental influence on bone remodeling [31, 32]. On one flank, NF‐κB acts as a primary catalyst for osteoclastogenesis, propelling precursor cells toward mature, bone‐resorbing phenotypes [33]. On the other, unchecked NF‐κB signaling cripples the differentiation and viability of bone‐forming osteoblasts [34, 35]. To dismantle these pro‐resorptive drivers, SIRT1 directly targets the p65 subunit for deacetylation [36]. Specifically, SIRT1 strips acetyl groups from the lysine 310 (K310) residue on p65. Because p65 acetylation typically fortifies DNA binding and accelerates target gene transcription to amplify inflammation [37], SIRT1‐mediated deacetylation curtails this transcriptional output, dropping the expression of both osteoclastogenic factors and inflammatory mediators [38, 39]. Cellular assays corroborate this mechanism, confirming that resveratrol treatment visibly drops acetylated NF‐κB levels [40] an effect entirely dependent on SIRT1, given that RNA interference‐mediated knockdown of SIRT1 abolishes resveratrol's anti‐inflammatory properties [40].

Far from theoretical, this NF‐κB blockade translates into a comprehensive disruption of the entire signaling cascade. Multiple points along the pathway yield to resveratrol‐driven SIRT1 action. For instance, it suppresses IκB kinase (IKK) activity, thereby halting the upstream phosphorylation required to mark the inhibitory protein IκBα for degradation [41, 42]. Because IκBα breakdown is mandatory for the liberation and nuclear translocation of active NF‐κB dimers, intercepting this phosphorylation traps NF‐κB safely within the cytoplasm, far from its nuclear DNA targets [37]. Even should minor fractions of NF‐κB breach the nuclear envelope, prior p65 deacetylation severely compromises their transcriptional competence [43]. This multi‐pronged assault clarifies why resveratrol so effectively neutralizes RANKL (Receptor Activator of NF‐κB Ligand)‐driven osteoclastogenesis, the dominant pathway for new osteoclast formation [41, 44, 45].

Beyond checking osteoclasts, the SIRT1‐NF‐κB axis safeguards osteoblasts against inflammation‐induced trauma. Pro‐inflammatory signals such as TNF‐α actively induce osteoblast apoptosis while suppressing differentiation, driving net skeletal depletion [35]. Overexpressing SIRT1 rescues murine osteoblasts from TNF‐α injury primarily through NF‐κB repression [35, 46]. Similarly, resveratrol reverses TNF‐α‐triggered apoptosis and restores the osteogenic differentiation capacity of bone marrow‐derived mesenchymal stem cells (MSCs). This dual capacity simultaneously throttling osteoclast destruction and preserving osteoblast formation forms the therapeutic bedrock of resveratrol's efficacy.

Operating within a broader molecular network, this core mechanism intersects with several parallel signaling pathways. Resveratrol stimulates SIRT1 expression while concurrently upregulating canonical Wnt/β‐catenin signaling, a pathway indispensable for osteoblast differentiation and bone mass accumulation [47]. Furthermore, it promotes osteogenesis by tuning the SIRT1/Runx2 axis, where SIRT1‐mediated deacetylation of the master transcription factor Runx2 enhances its functional potency [48, 49, 50]. Additional crosstalk involves the activation of the SIRT1/PI3K/AKT pathway in osteoporotic murine BMSCs alongside modulation of the SIRT1/FOXO3a cascade to strengthen resistance against oxidative stress [51].

Despite the compelling body of evidence supporting the SIRT1–NF‐κB axis, the strength and directness of mechanistic evidence vary across experimental models. While numerous in vitro and in vivo studies directly confirm SIRT1‐mediated deacetylation of NF‐κB p65, other investigations suggest that resveratrol's osteoprotective actions involve indirect associative mechanisms or parallel signaling crosstalk, such as the FoxO1/PI3K‐AKT, SIRT7, and Wnt/β‐catenin pathways. Furthermore, occasional discrepancies in the literature including reports where resveratrol failed to suppress osteoclastogenesis highlight the sensitive dependence of cellular outcomes on experimental variables. These inconsistent findings are largely attributable to differences in cell types (e.g., primary bone marrow cells vs. immortalized cell lines), narrow biphasic concentration windows (where low doses stimulate differentiation while high doses may exert cytotoxic or paradoxical effects), exposure durations, and specific disease models. Consequently, while the SIRT1–NF‐κB pathway represents a pivotal signaling axis, it should be viewed as a central node within a broader, highly context‐dependent regulatory network rather than a universally dominant, standalone mechanism. Nevertheless, ultimately these interconnected pathways demonstrate that SIRT1 activation by resveratrol orchestrates a synchronized cellular response, shifting the bone remodeling equilibrium toward enhanced structural integrity and elevated bone mass (Table 1).

**Table 1:** Studies on resveratrol–SIRT1–NF‐κB in bone disease.

Model/disease	SIRT1–NF‐κB mechanism	Bone/cell outcome	Key quantitative or directional finding	References
Bone‐derived cells; RANKL‐driven osteoclastogenesis	Resveratrol inhibited RANKL‐induced NF‐κB by suppressing IκBα phosphorylation, IκBα degradation, and IKK activity; SIRT1 associated with p300 and deacetylated NF‐κB	Prevented multinucleated osteoclast formation; promoted osteogenesis via Cbfa‐1/SIRT1	Complete inhibition of RANKL‐induced NF‐κB activation was reported in vitro	[37]
Human mesenchymal progenitors/osteogenesis	Resveratrol‐induced osteogenesis was mediated mainly through SIRT1, with SIRT1‐FOXO3A complex activating RUNX2; not a direct NF‐κB paper	Increased ALP activity and RUNX2 promoter activity	Sirtinol completely blocked resveratrol's osteogenic effect in the reported assay	[52]
OVX rat postmenopausal osteoporosis; BMSCs in vitro	Resveratrol activated SIRT1 and subsequently suppressed NF‐κB, with decreased p‐IκBα and NF‐κB p65	Improved osteoblast differentiation, bone structure, and BMD	BMD increased from 0.165 ± 0.050 in OVX to 0.205 ± 0.023 and 0.214 ± 0.053 g/cm^2^ at medium and high dose	[53]
Rat osteoporosis model	Resveratrol promoted SIRT1, suppressed NF‐κB, and activated IκBα expression	Improved BMD, femoral porosity, mechanical properties; lowered ALP and OC	Reported marked improvement in BMD and bone mechanics with lower ALP and osteocalcin	[54]
OVX rats and oxidative injury model	Mainly FoxO1/PI3K‐AKT oxidative‐stress mechanism; intersects upstream with osteoclast control rather than directly proving SIRT1–NF‐κB	Reduced osteoclastogenesis and bone loss	Resveratrol decreased RANKL and TRAP‐5b and increased OPG in OVX rats	[55]
Mouse BMM osteoclastogenesis	SIRT1 overexpression or resveratrol inhibited osteoclastogenesis; SIRT1 depletion had the opposite effect	Reduced TRAP activity, osteoclast markers, multinucleated cells	SIRT1 activation inhibited osteoclastogenesis, while EX‐527 or silencing promoted it	[56]
Aged female mice; osteoblast cell lines	Resveratrol positively regulated SIRT1, and SIRT1 deletion blocked RSV‐induced osteoblast activity; mechanism centered on SIRT1–eNOS–BMP2	Increased osteoblast activity and bone growth	Loss of SIRT1 blocked RSV‐induced osteoblast activity	[57]
Osteoporosis rats; DEX‐treated osteoblasts	Resveratrol increased SIRT1, LC3, Beclin‐1 and downregulated p‐AKT/p‐mTOR; focused on autophagy rather than direct NF‐κB	Protected osteoblasts and improved bone quality	High‐dose RSV increased SIRT1 activity and improved bone quality in osteoporotic rats	[58]
OVX mice; H2O2‐treated osteoblasts	Resveratrol upregulated SIRT1 and increased nuclear FoxO1; oxidative‐stress resistance mechanism	Promoted osteogenesis, proliferation, and survival; reduced apoptosis	Resveratrol ameliorated bone loss and promoted osteogenesis in OVX mice	[59]
MSC osteogenic differentiation with TNF‐β inflammatory suppression	Resveratrol reversed TNF‐β effects by activating SIRT1 and Runx2 and suppressing TNF‐β‐induced NF‐κB; SIRT1 knockdown blocked benefit	Restored osteogenic differentiation of MSCs	SIRT1 antisense or nicotinamide mimicked suppression, while RSV reversed it	[60]
Primary mouse BMCs and RAW264.7 osteoclastogenesis	Important caveat: in this ex vivo system, resveratrol did not inhibit osteoclastogenesis, unlike SRT2183/SRT1720	RSV failed to reduce osteoclast formation or resorption here	5 μM RSV did not reduce osteoclastogenesis and appeared to increase it in some assays	[46]
Steroid‐induced femoral head osteonecrosis	Resveratrol altered Sirt1, NF‐κB, and RANKL, partly via miR‐146a	Suppressed osteoclastogenesis and improved bone formation/microstructure	Resveratrol lowered osteonecrosis incidence and improved micro‐CT bone structure	[61]
OVX osteoporosis; BMSCs	Resveratrol acted through miR‐193a/SIRT7‐mediated NF‐κB signaling rather than SIRT1 specifically	Promoted osteogenic differentiation and alleviated osteoporosis	SIRT7 promoted osteogenic differentiation through NF‐κB signaling, and RSV acted through this axis	[62]
OVX rats; menopausal women cross‐sectional arm	Resveratrol increased femur SIRT1 and lowered RANKL; human arm linked low SIRT1 with low bone mass	Lower bone loss in rats; SIRT1 associated with bone mass in women	Femur SIRT1, β‐catenin, and BMD increased, while RANKL decreased in OVX rats given resveratrol	[47]
LPS‐stimulated RAW264.7 osteoclast precursor model	Resveratrol increased miR‐181a‐5p and reduced TRAF6/TAK1, p‐IκBα, and NF‐κB65	Reduced inflammatory cytokines, osteoclast differentiation, and bone resorption markers	TNF‐α, IL‐1β, IL‐6, p‐IκBα, p‐TAK1, NF‐κB65, and TRAF6 all decreased with RSV	[63]
Active vitamin D insufficiency mouse model; BM‐MSCs	Resveratrol attenuated osteoporosis via the Sirt1/PGC1α axis; NF‐κB not the main tested node	Promoted osteoblastic formation and reduced osteoclastic resorption	Resveratrol attenuated bone loss caused by vitamin D insufficiency	[64]

## 4 Therapeutic Efficacy in Osteoporosis

To comprehensively understand the therapeutic efficacy of resveratrol in skeletal pathologies, it is essential to evaluate its impact beyond the binary interaction of osteoblasts and osteoclasts, extending to the coordinated cellular network within the basic multicellular unit (BMU). As elegantly outlined by Kular et al., bone remodeling relies on an intimate, multi‐lineage cross‐talk involving osteoclasts, bone‐forming osteoblasts, mechanosensing osteocytes, bone lining cells, bone‐resident macrophages (osteomacs), and vascular endothelial cells [65]. The pathogenesis of osteoporosis and related skeletal disorders is fundamentally driven by the disruption of this multicellular communication network. Consequently, resveratrol's therapeutic potential stems from its capacity to restore systemic and local cellular homeostasis across this entire microenvironment. Furthermore, in alignment with the broader therapeutic paradigm of natural bioactive compounds possessing dual regulatory properties, ideal anti‐osteoporotic agents typically exhibit concurrent pro‐anabolic and anti‐catabolic effects [66]. Similar to other promising alternative botanical interventions, resveratrol bridges this gap by simultaneously stimulating osteoblast‐mediated bone formation and suppressing osteoclast‐driven bone resorption, making it a compelling candidate to restore the physiological bone remodeling equilibrium.

Characterized by microarchitectural deterioration and low bone mass, osteoporosis represents a systemic skeletal disease and stands as the most extensively investigated condition for resveratrol's therapeutic exploration. Backed by a robust mechanistic rationale originating from the SIRT1‐NF‐κB axis, this strategy finds powerful substantiation through an expansive body of preclinical research utilizing disease models. Specifically, ovariectomized (OVX) mice and rats faithfully replicate the estrogen‐depleted state responsible for postmenopausal bone loss in humans. Across numerous investigations, routine dietary supplementation with resveratrol consistently counters these pathological skeletal alterations. Micro‐computed tomography (micro‐CT) alongside histological evaluations confirm that treatment preserves bone mass, bolsters trabecular microarchitecture, and significantly elevates BMD [67, 68]. Histological and micro‐computed tomography (micro‐CT) analyses in OVX rat models have revealed positive effects on trabecular bone structure and mineral density [26, 68]. Ultimately, upregulation of SIRT1 expression coupled with the modulation of downstream pathways such as the stimulation of Wnt/β‐catenin signaling directly drives these protective phenomena [47, 69].

At the biochemical level, administering resveratrol in OVX models fosters a favorable, bone‐protective remodeling equilibrium. The compound promotes osteogenesis under low‐estrogen conditions while simultaneously checking runaway bone resorption [70]. Serum biomarker profiles frequently reflect this transition, displaying elevated bone formation markers alongside suppressed resorption metrics, though isolated discrepancies remain within the literature [53, 71, 72]. Crucially, long‐term administration successfully wards off ovariectomy‐induced osteopenia without triggering uterine hyperplasia, steering clear of a frequent adverse outcome associated with standard hormone‐replacement therapies [67]. These preclinical data converge on a singular insight: by driving the SIRT1‐NF‐κB signaling axis alongside allied protective networks, resveratrol effectively mitigates skeletal degradation in animal models [54].

A critical synthesis of current clinical investigations, however, reveals substantial heterogeneity that accounts for conflicting trial outcomes. Discrepancies across human studies stem from several key confounding variables, including baseline skeletal status (e.g., osteopenic vs. normal postmenopausal women), varying menopausal stages, divergent intervention durations (ranging from short‐term trials to multi‐year follow‐ups), broad dosage ranges (typically 150 to 1500 mg/day), differences in delivery formulations, and the profound pharmacokinetic limitations of native resveratrol bioavailability. Furthermore, variations in selected outcome measures such as regional BMD at the lumbar spine versus femoral neck versus systemic bone turnover markers further complicate direct comparisons. Consequently, current clinical evidence remains insufficient to establish an optimal therapeutic dose, define a precise target patient population, or confirm definitive clinical efficacy, underscoring the necessity for rigorously standardized, large‐scale clinical trials.

### 4.1 Role in Fracture Healing and Management of Inflammatory Bone Loss

Beyond its recognized utility in managing osteoporosis, the regenerative and anti‐inflammatory attributes of resveratrol orchestrated primarily through the SIRT1‐NF‐κB axis position it as a compelling agent for bolstering fracture healing and suppressing pathological bone deterioration driven by chronic inflammation. Repairing skeletal fractures is a convoluted biological cascade dependent upon an intricately sequenced progression of inflammatory, reparative, and remodeling stages [73]. Unfortunately, advanced age compromises this trajectory by distorting immune dynamics and elevating systemic inflammation, frequently culminating in delayed unions or fracture non‐unions [74]. Herein, resveratrol's regulatory influence on inflammatory pathways assumes critical importance. Within osteoporotic animal models inherently plagued by compromised fracture repair, resveratrol administration visibly enhances the biomechanical integrity and structural quality of newly formed calluses [75]. Such pro‐regenerative outcomes are undoubtedly multifaceted, encompassing the dampening of initial inflammatory cascades coupled with the stimulation of subsequent osteogenic phases [76].

Another vital therapeutic frontier involves combating inflammatory bone loss manifest in pathologies like spinal cord injury (SCI), periodontitis, and rheumatoid arthritis (RA). Persistent inflammation acts as the primary engine of tissue destruction in these disorders, fueled heavily by sustained RANKL and NF‐κB pathway activation [77, 78]. By shutting down these pathways, resveratrol delivers a direct countermeasure against inflammatory bone erosion. Within periodontitis a condition defined by inflammation‐mediated alveolar bone degradation resveratrol exhibits profound utility. Investigations reveal that it curtails localized inflammation and safeguards against alveolar bone loss, suppressing the underlying NF‐κB and RANKL‐driven osteoclastogenesis; indeed, phytochemicals like curcumin and resveratrol are widely recognized for mitigating inflammation‐linked bone decay in periodontal tissues [79].

Confronting severe disuse osteoporosis following SCI, resveratrol demonstrates notable efficacy by amplifying the protective mechanisms of calcium supplementation via modulation of the SIRT1/FOXO3a pathway to combat oxidative stress [51]. Glucocorticoid‐induced osteoporosis (GIO), a frequent comorbidity among chronic inflammatory disease patients dependent on long‐term steroid therapy, represents yet another promising application. Driven by simultaneously suppressed bone formation and accelerated resorption, GIO responds favorably to resveratrol's capacity to shield osteoblasts from inflammatory insults while inhibiting particle‐induced osteoclastogenesis [69, 80]. Moreover, resveratrol prevents skeletal degradation in ovariectomized rodent models by regulating microRNA‐338‐3p expression, thereby modulating the local inflammatory milieu [81].

Collectively, data across these diverse experimental frameworks confirm that resveratrol's SIRT1‐NF‐κB inhibitory framework extends broadly across various pathologies where inflammation drives skeletal loss. Intercepting this pivotal molecular hub allows resveratrol to neutralize a core disease driver, offering an intervention strategy distinct from standard anabolic or antiresorptive medications. Beyond inflammatory pathways, the accumulation of reactive oxygen species (ROS) within the bone microenvironment acts as a potent driver of osteoclast activation and bone resorption [82]. Under physiological and pathological stress, intracellular redox homeostasis is largely governed by the Keap1/Nrf2 signaling axis. Similar to other bioactive natural modulators that target redox balance in bone cells, resveratrol exerts its protective and antiresorptive effects partly by engaging the Nrf2/ARE antioxidant response pathway [83]. By suppressing intracellular ROS generation and interfering with redox‐sensitive signaling cascades in osteoclast precursors, resveratrol counteracts oxidative stress‐induced cellular damage and inhibits excessive osteoclastogenesis [41] (as highlighted in Table 2 for ROS‐scavenging interventions).

**Table 2:** Studies on resveratrol and bone repair.

Model/population	Bone context	Main mechanism/pathway	Key findings	Relevance to fracture healing or inflammatory bone loss	References
Human periosteum‐derived MSCs	Osteogenesis relevant to fracture healing	Mitochondrial biogenesis linked to osteodifferentiation	At 5 μM, resveratrol increased ALP, calcium deposition, mitochondrial mass, and mtDNA, while > 10 μM impaired proliferation and viability	Strongly relevant to fracture healing, because periosteal MSCs are key repair cells	[84]
Rat calvarial defects and tibial implants	Bone defect healing and implant integration	Upregulation of osteogenic genes	Resveratrol reduced remaining defect area, increased implant counter‐torque, and increased BMP‐2, BMP‐7, and OPN expression	Direct evidence for fracture‐like bone repair and biomechanical integration	[85]
OVX rats, isolated osteoblasts and osteoclasts	Estrogen‐deficiency bone loss	Autophagy regulation	Resveratrol dose‐dependently increased bone calcium and BMD, increased VEGF and COL1A1 in osteoblasts, reduced RANKL in osteoclasts, and reversed dysregulated autophagy	Supports inflammatory/resorptive bone loss management and osteoblast function	[86]
RAW264.7 cells, BMSCs, osteoporotic rat femoral condyle defects	Osteoporotic bone regeneration with inflammation	Local sustained release with anti‐inflammatory and osteogenic effects	Microspheres reduced TNF‐α, IL‐1β, and iNOS, upregulated Runx2, ALP, Col‐1, and OCN, and enhanced entochondrostosis and bone regeneration in vivo	One of the most direct studies for fracture healing under inflammatory/osteoporotic conditions	[87]
Senescent osteoblasts, BMSCs, macrophages, HUVECs	Elderly fracture‐healing impairment	Anti‐senescence, ROS scavenging, M2 polarization, angiogenesis	Encapsulated resveratrol improved bioavailability, reduced senescence and ROS, improved osteogenesis, promoted macrophage M2 polarization, enhanced angiogenesis, and outperformed free resveratrol	Strong mechanistic support for elderly fracture healing	[88]
BMSCs and rat cranial defects	Critical‐sized bone defect regeneration	Sustained release with osteoconduction and osteoinduction	Res‐SLNs/GelMA scaffolds improved BMSC osteogenic differentiation and bone regeneration in vivo; 0.02% scaffold performed best	Direct evidence for bone defect healing applications	[89]
Postmenopausal women, randomized placebo‐controlled crossover trial	Clinical bone loss at common fracture sites	Likely antiresorptive and perfusion‐related effects	Resveratrol increased lumbar spine and femoral neck BMD and reduced CTX‐1 by 7.24%; femoral neck T‐score improved and fracture risk estimates declined	Clinical support for bone loss management, but not direct fracture‐healing evidence	[72]

## 5 Translational Challenges and Drug Development Strategies

Compelling mechanistic rationales and robust preclinical validation notwithstanding, translating resveratrol into a widely adopted therapeutic hits a severe bottleneck. Its pharmacokinetic profile is fundamentally flawed. Poor oral bioavailability remains the supreme barrier to unlocking its clinical utility. Estimates of intestinal absorption in humans actually run quite high, capturing roughly 70%–75% of the ingested dose [19, 90, 91]. Yet, disaster strikes immediately thereafter. Extensive first‐pass metabolism decimates the compound within the gut wall and liver [92]. Driven predominantly by sulfation and glucuronidation, these conjugation cascades swiftly transform the biologically active parent molecule into inert sulfate and glucuronide metabolites [90, 93]. Intact resveratrol reaching the systemic circulation, therefore, represents a mere fraction of the initial load. Human trials peg this oral bioavailability at less than 1%, plunging occasionally to an abysmal 0.5% [19, 94, 95]. Murine models align closely with this rapid clearance, demonstrating a bioavailability threshold around 6% in rats [96]. Circulating plasma concentrations of the active parent compound thus remain vanishingly low, leaving conjugated derivatives to dominate the bloodstream [94, 97]. Achieving therapeutic tissue concentrations consequently demands massive oral doses. Such extreme regimens inevitably spark critical concerns regarding overall patient tolerability, sheer financial cost, and long‐term safety.

To circumvent these metabolic blockades, researchers have pioneered highly innovative drug delivery architectures. Protecting the payload from premature enzymatic destruction while prolonging systemic circulation forms the primary objective here. Nanoparticle platforms shine exceptionally bright in this arena [98]. Encapsulating the polyphenol within advanced nanocarriers including solid lipid nanoparticles, liposomes, or synthetic PLGA polymers successfully shields the active compound from devastating gut and hepatic enzymes [99, 100]. Shielding the payload in this manner dramatically elevates the proportion of intact resveratrol infiltrating the systemic blood supply. Sequestering resveratrol within casein‐derived nanoparticles, for instance, amplifies its calculated oral bioavailability tenfold over the naked polyphenol [99, 101]. Surpassing standard liposomal formulations, solid lipid nanocarriers frequently deliver superior chemical stability and enhanced overall therapeutic potency. Engineered meticulously for sustained release, these modern platforms stretch the therapeutic window. Dosing frequency drops accordingly [99].

Engineering bone‐targeted delivery vectors represents the definitive frontier in skeletal pharmacology. The clinical ambition is precise. Researchers seek to concentrate the botanical payload exclusively within osseous architecture, thereby amplifying localized efficacy while simultaneously blunting off‐target systemic exposure. Exploiting molecular affinity for hydroxyapatite the principal mineral constituent of the skeleton anchors this targeting strategy. Because standard bisphosphonates exhibit profound affinity for bone mineral surfaces, nanoparticles frequently undergo surface decoration with alendronate to hijack this homing mechanism [102, 103, 104]. Functionalizing the nanocarrier shell with this specific bisphosphonate yields a highly specialized vehicle programmed to bind bone mineral preferentially [104, 105]. Experimental assays definitively confirm that alendronate‐decorated constructs exhibit vastly superior binding affinity toward hydroxyapatite disks when measured against their non‐functionalized counterparts [104, 105]. Guided by this osteotropic homing, a dramatically expanded fraction of the encapsulated resveratrol payload deposits directly into the bone microenvironment to enact its vital osteoprotective functions. Recent comprehensive reviews enthusiastically underscore the immense clinical trajectory of these targeted nanoparticle platforms for conquering osteoporosis and allied skeletal pathologies [106, 107].

While still largely in the preclinical stage, these advanced delivery technologies represent a viable and promising solution to the primary hurdle facing resveratrol, paving the way for its potential clinical application at more effective and safer doses (Figure 3).

**Figure 3:**
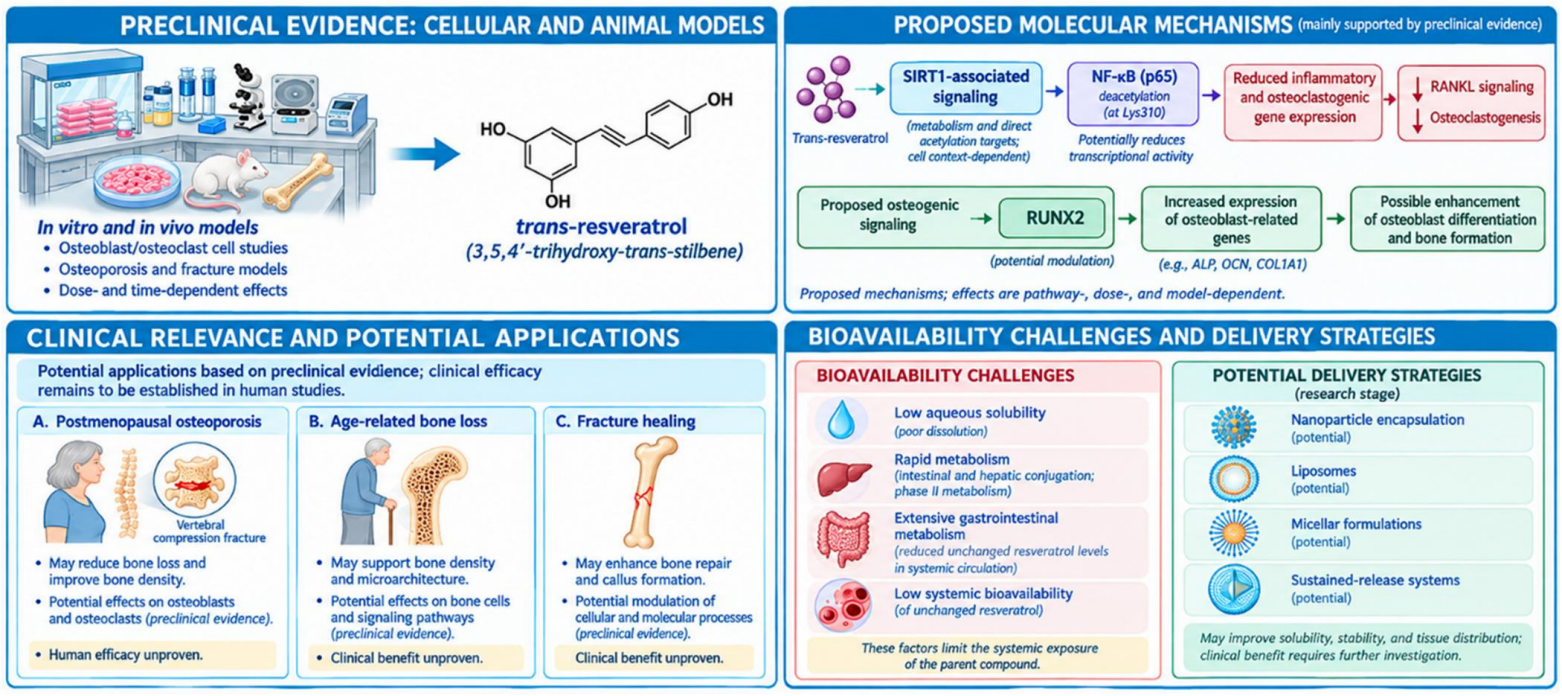
Preclinical evidence, proposed molecular mechanisms, potential clinical applications, and bioavailability challenges of trans‐resveratrol in bone health. Schematic overview of preclinical investigations of trans‐resveratrol in cellular and animal models, highlighting its potential modulation of SIRT1‐associated signaling, NF‐κB p65 deacetylation, inflammatory responses, osteoclastogenesis, and RUNX2‐associated osteogenic pathways. Potential applications in postmenopausal osteoporosis, age‐related bone loss, and fracture healing are illustrated, although clinical efficacy in humans remains to be established. Major pharmacokinetic challenges, including low aqueous solubility, extensive intestinal and hepatic metabolism, and low systemic exposure to unchanged resveratrol, are presented alongside emerging delivery strategies, such as nanoparticle encapsulation, liposomes, micellar formulations, and sustained‐release systems. The biological effects of resveratrol depend on the experimental model, dose, and exposure conditions, and the clinical benefits of targeted delivery approaches require further investigation (Schematic created by the authors using vector graphic software).

## 6 Current Status and Future Prospects in Clinical Practice

Emerging as a compelling natural agent for skeletal maintenance, resveratrol boasts preclinical backing those points toward potential osteoporosis prevention driven by SIRT1 activation and NF‐κB suppression. By promoting osteoblast differentiation while concurrently throttling osteoclastogenesis, this polyphenol shifts the bone remodeling balance toward net tissue accumulation. Protection against oxidative stress‐induced apoptosis in bone cells stems directly from SIRT1‐driven pathways that amplify antioxidant defenses and repress inflammatory gene expression. Yet, the current clinical standing of resveratrol remains that of an unvalidated therapeutic. While standard pharmaceutical options like bisphosphonates exhibit robust, trial‐proven efficacy in elevating BMD and slashing fracture rates [108], human data for resveratrol stay stubbornly inconsistent. Conflicting trial outcomes regarding BMD preclude its endorsement as a standard‐of‐care replacement for established therapies [109].

Clinical irrelevance does not follow, however. Boasting a favorable safety profile alongside pleiotropic advantages, resveratrol occupies a distinct niche as a complementary or nutraceutical intervention. For postmenopausal women and other individuals aiming to safeguard skeletal integrity, it offers a scientifically grounded choice. Notably, a landmark randomized controlled trial demonstrated that a 150 mg daily dosage successfully decelerated bone loss across the femoral neck and lumbar spine, establishing a concrete reference point for interested patients [72]. Provided clinicians frame these recommendations carefully transparently managing expectations and acknowledging current evidence limitations resveratrol serves well in this supportive capacity.

Looking ahead, the orthopedic trajectory of resveratrol is tightly bound to innovations in drug delivery engineering. Abysmal oral bioavailability remains the primary bottleneck restricting systemic distribution [90, 95]. Emerging technologies like bone‐targeted nanoparticles promise to rewrite this narrative by channeling therapeutically active concentrations directly into the skeletal microenvironment [106, 107]. Should these delivery platforms transition successfully into human medicine, resveratrol could step forward as a legitimate clinical asset acting either as an independent intervention for mild osteopenia or as an adjunctive partner to conventional drugs. Ultimately, transforming this botanical compound into a mainstream therapy hinges entirely on conquering its pharmacokinetic limits. Backed by sustained nanotechnology investment and rigorous clinical trials, resveratrol maintains the immense promise of bridging mechanistic brilliance with practical healing to preserve human bone health.

## 7 Conclusion

Preclinical evidence suggests that resveratrol can influence bone remodeling by promoting osteoblast activity and inhibiting osteoclastogenesis through multiple signaling pathways, including SIRT1‐associated mechanisms. However, clinical evidence remains limited and heterogeneous, and current data are insufficient to establish resveratrol as a standard therapeutic intervention for osteoporosis. Further well‐designed, adequately powered clinical trials are required to clarify its efficacy, optimal dosing, long‐term safety, and clinically meaningful effects on bone density and fracture risk.

Driven primarily by the SIRT1‐mediated deacetylation of the NF‐κB p65 subunit, these processes suppress inflammatory gene transcription and damp down RANKL‐driven osteoclast development, alongside boosting osteogenesis via the deacetylation of Runx2 and beta‐catenin. Furthermore, Nrf2‐dependent activation of antioxidant defenses provides critical shielding against oxidative stress‐induced apoptosis in bone cells.

Notwithstanding consistent protection against skeletal degradation across animal models, translating these insights to human medicine demands rigorous further validation. While select clinical trials point toward favorable impacts on BMD and turnover biomarkers among postmenopausal women, inconsistent human outcomes underscore an urgent requirement for extended, large‐scale trials deploying standardized formulations. Meanwhile, persistent bioavailability hurdles are actively targeted by novel structural analogs and advanced carrier technologies. As research progresses, resveratrol may well secure a vital place within holistic bone health regimens functioning either as a standalone nutraceutical or an adjunctive partner to standard treatments. Moving forward, scientific efforts must prioritize formulation optimization, the identification of responsive patient cohorts, and definitive clinical testing to fully define resveratrol's therapeutic utility in safeguarding skeletal health amid a rapidly aging global population.

## Author Contributions

Seyyed Hossein Khatami, Faezeh Gouhari, Alireza Manafi‐Rasi, Maryam Fallah, Sajad Ehtiati, Ahmad Reza Ahmadi Abdashti, Mohsen Ariannik, Mina Fakhrzadegan, Shahin Talebi, Diba Rafienejad, Parasta Heidari, and Reza Zandi contributed to data collection and article writing. Figures were illustrated by Seyyed Hossein Khatami and Sajad Ehtiati. The study was conceptualized and designed by Seyyed Hossein Khatami and Reza Zandi. Reza Zandi was also responsible for project administration and supervision. All authors read and approved the final version of the article.

## Funding

The authors have nothing to report.

## Ethics Statement

The authors have nothing to report.

## Conflicts of Interest

The authors declare no conflicts of interest.

## Data Availability

Data sharing not applicable to this article as no datasets were generated or analysed during the current study.
